# Dr. John Park Mason

**Published:** 1910-08

**Authors:** 


Dr. John Park Mason, of Elmira, N. Y., formerly of Houghton, Mich., died at his home July 4, 1910, aged 56 years. He graduated at the University of Buffalo, medical department, in 1880, after several years at Cornell University where he was a noted athlete, having been the captain and stroke of the winning crew at Saratoga in 1876. Upon taking his medical degree he became physician to the great mining company in upper Michigan, practising with success in that capacity, and also in private practice. Several years ago he suffered injury from a fall which developed finally into cerebral disturbance, from which he never fully recovered, and in consequence he retired from practice and business, having accumulated a comfortable fortune.

